# Gene Polymorphisms of Glutathione S-Transferase T1/M1 in Egyptian Children and Adolescents with Type 1 Diabetes Mellitus

**DOI:** 10.4274/jcrpe.3690

**Published:** 2017-06-01

**Authors:** Naglaa Barseem, Mona Elsamalehy

**Affiliations:** 1 Menoufia University Faculty of Medicine, Department of Pediatrics, Shibin Elkom, Egypt; 2 Menoufia University Faculty of Science, Department of Chemistry, Shibin Elkom, Egypt

**Keywords:** Glutathione S-transferase T1 and M1, Gene polymorphisms, type 1 diabetes mellitus

## Abstract

**Objective:**

Oxidative stress plays an important role in the pathogenesis of type 1 diabetes mellitus (T1DM). To evaluate the association of glutathione S-transferase mu 1 (GST M1) and glutathione S-transferase theta 1 (GST T1) polymorphisms with development of T1DM and disease-related risk factors.

**Methods:**

Measurement of fasting glucose, serum creatinine, lipid profile, and glycosylated hemoglobin (HbA1c), as well as evaluation of GST T1 and M1 genetic polymorphisms using polymerase chain reaction were done in 64 diabetic children and 41 controls.

**Results:**

The diabetic group had significantly higher fasting glucose, HbA1c, and cholesterol levels. GST T1 null genotype was more frequent in the diabetic than the control group with 4.2-fold increased risk of T1DM (odds ratio=4.2; 95% confidence interval=1.6-11.5; p=0.03). Significant positive associations were found with lipid profile, HbA1c, and duration of illness but not with age, age at onset, and body mass index.

**Conclusion:**

Gene polymorphisms of the enzyme GST are associated with development of T1DM and disease-related risk factors.

## What is already known on this topic?

Oxidative stress plays an important role in the pathogenesis of type 1 diabetes mellitus (T1DM). Control of diabetes throughout childhood and adolescence is mandatory.

## What this study adds?

To our knowledge, this is the first study on the association between glutathione genetic polymorphism and T1DM in Egypt. Glutathione S-transferase T1 null/M1 wild genotype can be strongly added to the predictive markers of the disease-related risk and complications.

## INTRODUCTION

Type 1 diabetes mellitus (T1DM) is the most common metabolic disorder in which both genetic and environmental factors are involved (1). T1DM is considered a chronic immune-mediated disorder. It was hypothesized that whilst children have a genetic predisposition to T1DM, there is likely to be an environmental factor that triggers the development of T1DM. Possible triggers that have been suggested include viral infection, vaccines, low levels of vitamin D, and cow’s milk (2). Oxidative stress is one of the important pathways that have been involved in the etiopathogenesis of T1DM (3). Complications of T1DM could be due to the cellular metabolism leading to hyperglycemia and excessive production of reactive oxygen species (ROS). ROS are a substantial threat to the human cells in children with T1DM. Many antioxidants are produced by human cells that counter the effects of these oxidants by decreasing their accumulation. One of the important antioxidants that protects against cellular damage caused by environmental toxins and accumulation of ROS is glutathione (GSH). ROS and xenobiotics are neutralized by GSH via glutathione S-transferase (GST); this enzyme converts these compounds into water-soluble compounds that can be easily eliminated (4,5). The human GSTs are a family of enzymes for neutralizing free radicals. They cause detoxification of electrophiles via glutathione conjugation (6). The loci encoding the GST enzymes are located on at least seven chromosomes. This multigene family included seven families (Alpha, Mu, Pi, Theta, Sigma, Zeta, and Omega). There has been substantial interest in studying the associations between particular allelic variants and altered risk of a variety of diseases. Several GST polymorphisms have been associated with an increased or decreased susceptibility to several diseases. Two of the important members of the GST family, named GST mu 1 (GST M1) and GST theta 1 (GST T1) have different polymorphisms. Persons with homozygous deletions of either the GST M1 or the GST T1 locus have no enzymatic activity of the respective enzyme (7,8).

This study aimed to evaluate the association of GST M1 and GST T1 polymorphisms with development of T1DM and disease-related risk factors.

## METHODS

The study included 64 diabetic children with T1DM with a mean age of 11.7±3.6 years; 26 boys and 38 girls. They were patients attending the Pediatric Genetic and Endocrinology Unit and the Pediatric Outpatient Clinic of Menoufia University Hospitals in Egypt. The study was conducted in the period from January 2015 to March 2016. Diagnosis of T1DM patients was based on the American Diabetes Association (ADA) criteria (9). Patients were followed up, regularly checked and investigated for diabetic complications as well as their current treatment regimens. Cases with type 2 diabetes and those with other chronic diseases such as hypothyroidism, hyperthyroidism, or hypoadrenalism were excluded.

Forty-one apparently healthy children of matched age and sex served as a control group. Written informed consent was obtained from each child included in the study or their participant parents. Ethical clearance was obtained for the research project. The study protocol conforms to the ethical guidelines of the 1964 Declaration of Helsinki and its later amendments. Data about the duration of illness and onset of the disease in children with T1DM were obtained from the parents. Body weight, height, and body mass index (BMI) were measured in each child. Biochemical parameters as fasting blood glucose, 2-hour postprandial (2hPP) glucose, serum creatinine, alanine aminotransferase activity, glycosylated hemoglobin (HbA1c), cholesterol, triglyceride levels were determined in each child. The patients were classified as having good or poor glycemic control according to the ADA criteria. The target age-specific HbA1c were as follows: 7.5%-8.5% in <6 year olds, ≤8% in children between 6 and 12 years, and ≤7.5% in those between 13 and 18 years of age (10).

### DNA Extraction and Genotype Determination

Genomic DNA was extracted from peripheral venous blood using Bio-spin whole blood genomic DNA extraction kit “Bioflux”, according to the recommended protocol. Screening for GST T1 and M1 deletion polymorphisms was done using the polymerase chain reaction (PCR) technique. Details of primers sequence are listed below.

### For GST M1 polymorphisms:

5´–GAACTCCCTGAAAAGCTAAAGC–3´ and 5´–GTTGGGCTCAAATATACGGTGG–3´.

### For GST T1:

5´–TTCGTTACTGGTCCTCACATCTC–3´ and 5´–TCACGGGATCATGGCCAGCA–3´.

To avoid false-negative readings, internal control was used for amplification of exon 7 of the *CYP1A1* gene, with primers sequence as 5´–AACTTCATCCACGTTCACC–3´and 5´–GAAGAGCCAAGGACAGGTAC–3´.

**Figure 1:**
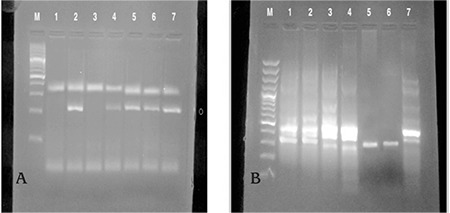
Polymerase chain reaction analysis of glutathione S-transferase M1/T1 gene polymorphism of products visualized in 2% agarose gel electrophoresis. (A) Lane M is a 100 bp DNA ladder, lanes 1,3 represent glutathione S-transferase M1 null, lanes 2,4,5,6 represent glutathione S-transferase M1 present (218 bp), lane 7 is a positive control sample. (B) Lane M is a 100 bp DNA ladder, lanes 5,6 represent glutathione S-transferase T1 null, lanes 1,2,3,4 represent glutathione S-transferase T1 present (480 bp), lane 7 is a positive control sample. *Note: Exon7 CYP1A1 appeared as internal control in all lanes at (315 bp).*

PCR products were electrophoresed in an agarose gel and visualized after ethidium bromide staining that yield bands of 218 bp, 480 bp, and 315 bp for GST M1, GST T1, and *CYP1A1*, respectively (Figure 1). The GST T1 or GST M1 genotype groups included homozygous and heterozygous states of that functional allele (11).

### Statistical Analysis

Data were collected, processed, and analyzed using SPSS (version 20) software. Continuous variables were presented as mean ± standard eror, and categorical ones were presented in percentage form. Chi-square and Fisher exact tests were used to assess the relationships between various demographic and disease-related risk factors and controls. The strength of association between GST genetic polymorphisms and development of DM was estimated by odds ratio (OR) and 95% confidence interval (CI). Logistic regression analysis was applied for checking calculated OR adjusted for independent variables and risk of T1DM. For all analyses, p-value of <0.05 was considered statistically significant.

## RESULTS

Mean duration of diabetes of the patients was 6.3±4.1 years. Of these diabetic children, 22 were 15-year-old or older; their Tanner stages were 2 or more. 29 of the 64 T1DM patients were in good glycemic control. Demographic and clinical data are shown in Table 1. The frequency rates of GST T1 null genotype were 42.2% and 14.6% in diabetic children and controls, respectively. This demonstrated a significantly increased risk of T1DM among our diabetic patients (OR 4.2, 95% CI 1.6-11.5, p-value=0.03). The frequency of GST M1 genotypes showed no significant difference. Double analysis of GST genotypes showed that GST T1 null/ M1 wild genotype was significantly more frequent in the diabetic group as compared to the control group (29.7% vs. 14.6%, respectively). This combination of alleles 3.2 times increased the risk of T1DM (OR=3.2; 95% CI=1.1-9χ^2^=10.6; p=0.014). There were no statistically significant differences regarding GST T1 null/M1 null genotype and other genotype combinations (Table 2). A significant difference (p<0.05) existed in duration of disease, fasting blood glucose, and HbA1C in relation to GST T1 null genotype, as compared to insignificant difference with age, age at onset, and BMI (Table 3).

Table 4 shows adjusted odd ratios for disease-related characteristics estimated by multivariate logistic regression analysis, which showed that patients with disease duration 5 y or more were more vulnerable to poor glycemic control (OR=1.07, p=0.004). This was also noticed among patients with raised serum TG level (OR=2.7, p=0.001). Those with GST T1 null/M1 present genotype had 2.2 times increased risk of impaired control of T1DM. Sex, age, and BMI were not significant factors of glycemic control.

## DISCUSSION

DM is associated with a high endogenous inflammatory load and oxidative stress (12). Environmental toxicants and oxidative stress are important in the development of T1DM. GSTs can enhance or decrease the toxicity of several compounds and robust GST activity depletes GSH, which can lead to the disruption of cellular homeostasis and cell death. GST T1 and M1 polymorphisms were supposed to be associated with many disorders like hypertension, ischemic heart disease, cancer, and allergic conditions (13). GSTs are involved in the detoxification of ROS and in the synthesis of different inflammatory mediators. Both mechanisms can lead to pancreatic beta cell damage. So, it is hypothesized that GST polymorphisms may have a role in the pathogenesis of T1DM. In our study, the associated risk between GST and T1DM was investigated. Patients with T1DM had a higher frequency of GST T1 null genotype than controls. This represented a significantly increased risk of T1DM (4.2-fold). The combination of GST T1 null/ M1 *wild* genotype was significantly more frequent in diabetic patients than controls. This represented a significantly increased risk of T1DM (3.2-fold). Our findings are concordant with many previous studies on GST T1/M1 gene polymorphisms that reported an increased risk of T1DM with null genotype (14,15,16,17). This can be attributed to the fact that carriers of GST null genotype have significantly lower antioxidant enzymatic activity (18). However, in 2005, Bekris et al (4) reported that null genotype is a protective gene regarding the risk of T1DM. The difference from our study can be explained by several possible mechanisms such as up-regulation of other antioxidant genes like superoxide dismutase gene that follows the depletion of GST activity. However, these interpretations require confirmation by further research. Previous studies reported that GST T1 or M1 gene polymorphisms can be risk factors for the development of diabetes mellitus and chronic diabetic complications (11,19). However, the reported results differ according to the authors, population, and the locality. Most of the reported data pertain to adult subjects with T2DM (14,20). Very limited studies were conducted on GST polymorphisms and diabetes susceptibility in patients with T1DM. Regarding GST T1 genotype, no association was found in a young Swedish population. However, M1 *wild* genotype was associated with a higher risk of T1DM in a 14-20-year-old group (6). In a Slovakian population, GST T1 null/M1 *wild* genotype was shown to increase the risk of T1DM (17). The differences in the results between the two populations can be attributed to ethnic background differences in the two populations. It is possible that, as noted in our study group of diabetic children, decreased antioxidant enzymatic activity due to GST T1 null allele, together with one of the tentative potentials due to GST M1 wild allele, constitutes a risk factor for T1DM. Results of other studies investigating the associations of GSTM1 and GSTT1 polymorphisms with T1DM suggest that the GST M1 null genotype is associated with T1DM protection and T1DM age-at-onset and that susceptibility to T1DM may involve GST conjugation (6). Also in our study, it was found that clinical and biochemical parameters showed a significant association with lipid profile, duration of illness, and glycosylated hemoglobin but not with age, age of onset, or BMI. Our results were in concordance with those reported by Vats et al (21). Studies on the impact of genotype on T1DM progression and development of its complications are rare. Hovnik et al (22), considering that the earliest signs of diabetes complications occur after 5-10 years and the highest incidence (between 75-95%) is observed after 10 years, stated that the duration of diabetic state is an important risk factor in development of microangiopathic complications in most patients with T1DM. However, a limited period of poor glycemic control can have a prolonged effect on disease complications. This effect, known as metabolic memory, has been demonstrated in the Epidemiology of Diabetes Interventions and Complications cohort follow-up, a study which showed that the GST T1 deletion, disease duration, and raised triglycerides independently show the risk of T1DM progression and glycemic control above the target level for age (23).

To the best of our knowledge, this is the first study on GST genetic polymorphisms and their relationship to T1DM risk among Egyptian children. Our results show the association of GSTT1 null/M1 present genotype with T1DM and indicate that testing of genetic markers in the glutathione family alone or in combination with other disease-associated factors can be used as a marker to predict the risk of T1DM. For the implementing of measures towards effective diabetes control, there is a need for larger scale studies which also involve interaction of complex pathologic effects.

## Figures and Tables

**Table 1:**
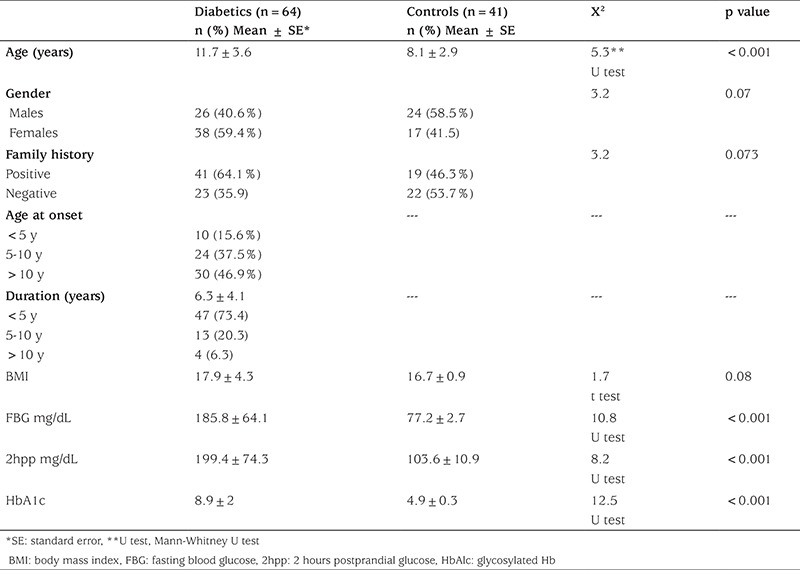
Demographic and laboratory data in the study and control groups

**Table 2:**
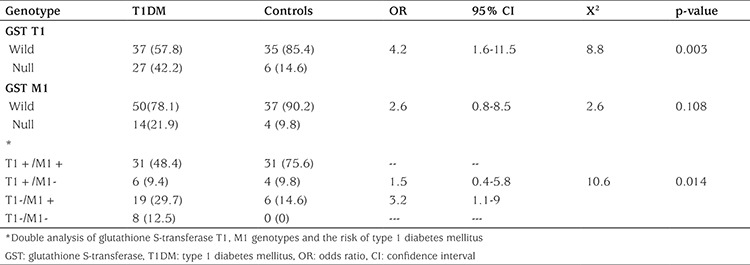
Association of glutathione S-transferase T1 and M1 genotypes with type 1 diabetes mellitus

**Table 3:**
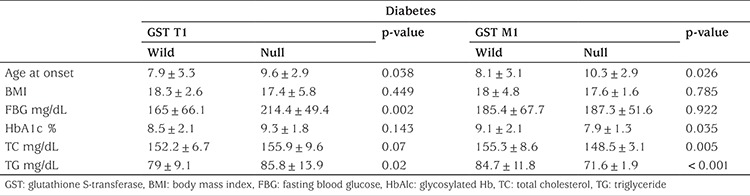
Relationships between glutathione S-transferase genotype and disease-related characters in diabetic patients

**Table 4:**
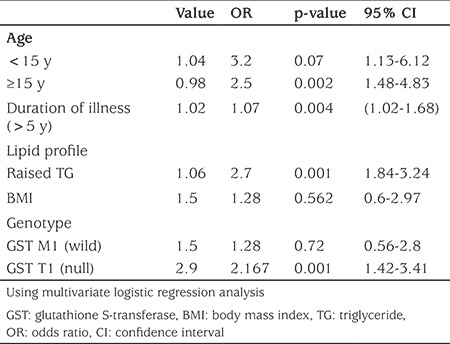
Adjusted odds ratio for disease-related characters among poorly controlled diabetic patients
